# CSN6–TRIM21 axis instigates cancer stemness during tumorigenesis

**DOI:** 10.1038/s41416-020-0779-9

**Published:** 2020-03-30

**Authors:** Baifu Qin, Shaomin Zou, Kai Li, Huashe Wang, Wenxia Wei, Boyu Zhang, Lishi Xiao, Hyun Ho Choi, Qin Tang, Dandan Huang, Qingxin Liu, Qihao Pan, Manqi Meng, Lekun Fang, Mong-Hong Lee

**Affiliations:** 1 Guangdong Provincial Key laboratory of Colorectal and Pelvic Floor Disease, The Sixth Affiliated Hospital of Sun Yat-sen University, 510655 Guangzhou, China; 2 Guangdong Research Institute of Gastroenterology, The Sixth Affiliated Hospital of Sun Yat-sen University, 510655 Guangzhou, China; 3 Department of Colorectal Surgery, The Sixth Affiliated Hospital of Sun Yat-sen University, 510655 Guangzhou, China

**Keywords:** Cancer stem cells, Colorectal cancer, Ubiquitylation

## Abstract

**Background:**

Cancer stem cells (CSCs) are responsible for tumour initiation, metastasis and recurrence. However, the mechanism of CSC formation, maintenance and expansion in colorectal cancer (CRC) remains poorly characterised.

**Methods:**

The role of COP9 signalosome subunit 6 (CSN6) in regulating cancer stemness was evaluated by organoid formation and limited dilution analysis. The role of CSN6–TRIM21–OCT1–ALDH1A1 axis in CSC formation was evaluated in vitro and in vivo. The association of CSN6, TRIM21 and ALDH1A1 expression was validated by a tissue microarray with 267 CRC patients.

**Results:**

The results showed that CSN6 is critical for sphere formation and maintaining the growth of patient-derived organoids (PDOs). We characterised the role of CSN6 in regulating cancer stemness, which involves the TRIM21 E3 ubiquitin ligase, transcription factor POU class 2 homeobox 1 (OCT1) and cancer stem cell marker aldehyde dehydrogenase 1 A1 (ALDH1A1). Our data showed that CSN6 facilitates ubiquitin-mediated degradation of TRIM21, which in turn decreases TRIM21-mediated OCT1 ubiquitination and subsequently stabilises OCT1. Consequently, OCT1 stabilisation leads to ALDH1A1expression and promotes cancer stemness. We further showed that the protein expression levels of CSN6, TRIM21 and ALDH1A1 can serve as prognostic markers for human CRC.

**Conclusions:**

In conclusion, we validate a pathway for cancer stemness regulation involving ALDH1A1 levels through the CSN6–TRIM21 axis, which may be utilised as CRC molecular markers and be targeted for therapeutic intervention in cancers.

## Background

Colorectal cancer (CRC) is the third most common cancer, with a high mortality rate.^1,2^ Although CRC patients benefit from adjuvant therapy, including chemotherapy and radiotherapy, after surgery, the rate of recurrence is still very alarming.^1^ Colon cancer stem cells (CSCs) are observed in CRC, and these cells have the properties of multilineage potential and self-renewal.^3–5^ Several studies have indicated that CSCs are responsible for tumour initiation,^6,7^ growth,^8^ metastatic spread,^9,10^ relapse and recurrence.^11^ Furthermore, emerging evidence shows that chemotherapy and radiotherapy increase CSC populations. In addition, progress in metagenome-wide association studies on faecal samples has identified microbial markers of CRC.^12,13^ Emerging evidence has demonstrated that dysbiosis of the gut microbiota^14^ can alter numerous aspects of cancer stemness regulation, resulting in the pathogenic processes of CRC. However, the understanding of CSC formation, maintenance and expansion remains poorly characterised.^15^

COP9 signalosome subunit 6 (CSN6) is one of the eight subunits of the COP9 signalosome that is involved in proteasome-mediated ubiquitination,^16,17^ cell cycle,^18,19^ transcriptional activation,^20^ signal transduction^17,19^ and tumorigenesis.^17,21^ Previous studies have shown that CSN6 is highly expressed in many tumours, including cervical cancer,^22^ CRC,^23^ breast cancer^24^ and T cell leukaemia.^25^ In addition, CSN6 regulates tumour growth^19,22^ and tumour metastasis^22,23^ in cancer. In CRC, high CSN6 expression leads to poor recurrence-free survival.^23^ As CSCs are able to self-renew and initiate tumour growth upon transplantation and ultimately promote tumour relapse and metastasis,^26^ these observations raise the following two questions: Does CSN6 regulate cancer stemness to promote tumour recurrence? What is the mechanism by which CSN6 overexpression triggers the cancer initiation (cancer stemness) process? To begin to address these questions, we ablated CSN6 expression in cultured cells and conducted sphere formation and patient-derived organoid (PDO) proliferation assays to investigate the role and mechanism of action of CSN6 in cancer stemness in vivo. These studies led to the discovery of a previously uncharacterised link between CSN6 and tripartite motif-containing protein 21 (TRIM21) and a crucial role for CSN6 in promoting cancer stemness.

In this study, we found that loss of CSN6 results in a significant decrease in sphere formation and PDO proliferation. Multiple lines of evidence indicate that CSN6 promotes the gene expression of stemness marker aldehyde dehydrogenase 1 A1 (ALDH1A1), indicating that CSN6 expression initiates cancer stemness in CRC. We further demonstrated that CSN6 facilitates TRIM21 autoubiquitination at the K214 and K217 sites, thereby reducing ubiquitination and degradation of OCT1, a transcription factor for the cancer stemness marker ALDH1A1. Thus CSN6 regulates CRC stemness by decreasing TRIM21 E3 ubiquitin ligase activity to stabilise OCT1 in order to increase the expression of ALDH1A1. These findings provide important insight into the new role of CSN6 in cancer stemness-initiating activities during tumorigenesis.

## Methods

### Cell culture and transfection

HEK293T and the human colon cancer cell lines including HCT116, DLD-1 and HCT-8 were obtained from ATCC. DLD-1 and HCT-8 cells were cultured in RPMI 1640 medium supplemented with 10% (v/v) foetal bovine serum (FBS) at 37 °C in 5% CO_2_. HEK293T and HCT116 cells were grown in Dulbecco’s modified Eagle’s medium (DMEM) with 10% FBS. Cells were plated in 6-well plate at a density of 2 × 10^5^ cells/well. Twenty-four hours later, cells were transfected with the indicated plasmids using Lipofectamine 2000 (Invitrogen) according to the manufacturer’s instructions.

Human *Csn6*, *Trim21* and *Oct1* genes was subcloned into pCMV5 to generate constructs with a Flag-tag or haemagglutinin (HA)-tag or into pCDNA3.1 to encode an Myc-tagged sequence. The human *Cul1* gene was subcloned into pCDNA3.1 to generate constructs with HA-tag or Myc-tag. Mutants pCMV5-Flag-TRIM21-K214R, pCMV5-Flag-TRIM21-K217R and pCMV5-Flag-TRIM21-K214R/K217R constructs were generated by using a Fast Mutagenesis Kit V2 (Vazyme) according to the manufacturer’s instructions. The N- or C-terminal of CSN6 was constructed into pCMV5-HA, while the N- or C-terminal of TRIM21 was constructed into pCMV5-Flag.

### Viral transduction, migration and invasion assay

To prepare lentivirus for protein expression, HEK293T cells were transfected with PLVX vectors and the packaging vectors PSPAX2 and pMD_2_G using polyethylenimine (Polysciences, 24765). Medium containing the virus was collected 24 and 48 h after transfection. HCT116 or DLD-1 cells were infected with the collected virus supernatant in the presence of polybrene (Millipore, TR-1003-G).

To prepare lentivirus for the knockdown of CSN6, the pGIPZ control was generated with the control oligonucleotide CTTCTAACACCGGAGGTCTT. pGIPZ CSN6 short hairpin RNA (shRNA) was generated with the shCSN6–1: CTTGAGAGAAACCGCTGTCAT and shCSN6–2: CAGTTTGTGAACAAGTTCAAT oligonucleotides targeting the CSN6 transcript.

For the migration and invasion assay, 24-well Boyden chambers (Corning, NY) were used and Matrigel (BD) were used for estimating cell invasion, and 1 × 10^5^ cells (for migration) or 1.5 × 10^5^ cells (for invasion) in 200 ml of serum-free media were seeded into upper chambers. RPMI 1640 supplemented with 10% FBS was placed in the lower chamber. Migration and invasion were scored at 12 and 24 h, respectively. Cells were fixed in 3.7% formaldehyde for 5 min at room temperature, stained with crystal violet for 15 min and counted under microscopy.

### Sphere-formation assay and limited dilution analysis

HCT116 or DLD-1 cells carrying scrambled or CSN6-specific shRNA were dissociated into a single-cell suspension. Then DLD-1, HCT116 and HCT-8 cells were cultured in 96-well Ultra-low Attachment surface plate with serum-free DMEM/F12 medium containing B27 supplement, 20 ng/ml epidermal growth factor (EGF) and 20 ng/ml basic fibroblast growth factor for 12 days. The sphere numbers in each well were quantified.

For limited dilution in vitro,^27^ the single DLD-1 cell suspension carrying scrambled or CSN6-specific shRNA was serially diluted to different doses, then seeded into 96-well plate with a final concentration of 100, 50, 10 and 2 cells/well. Twelve days later, wells containing spheres were counted and the sphere-formation frequency was calculated using the ELDA software.

For limited dilution in vivo,^27^ DLD-1 cells were transduced with control (scrambled shRNA) or shCSN6 (CSN6-specific shRNA) lentivirus for two times. For anaesthesia, the mice that were allocated to the experimental groups with randomisation were injected with 50 mg/kg pelltobarbitalum natricum by intraperitoneal injection. Then cells were dissociated into a single-cell suspension and injected subcutaneously into the 4-week-old female specific pathogen-free BALB/c nude mice (18–22 g) in a limited dilution series (1 × 10^6^, 5 × 10^5^, 1 × 10^5^ and 5 × 10^4^ cells/mice); each group have 6 mice (*n* = 6) and total number of experimental mice were 48. Xenografted mice were sacrificed by CO_2_ inhalation to observe tumour formation and tumour growth curve when the tumour volume reached about 500 mm^3^ or the mice were monitored for 70 days. All animal work was administered according to the guidelines of Institution Animal Care and Use Committee and all the protocols were approved by the Sixth Affiliated Hospital of Sun Yat-sen University. The number of ethical approval was 20181123-002.

### Real-time quantitative PCR (qPCR)

Total RNA was extracted with TRIzol (Invitrogen) and reverse transcribed using ReverTra Ace® qPCR RT Master Mix with gDNA Remover (TOYOBO). Real-time PCR was performed using the 2× SYBR Green qPCR Master Mix (bimake) in a LightCycler® 480 II (Roche) instrument. The qPCR primers are shown in Table S1.

### Organoid culture

Human CRC organoid culture was performed as previously described.^28^ Fresh CRC surgical specimens were washed with cold phosphate-buffered saline (PBS) and cut into small pieces, then digested with EDTA. After digesting into clumps of cells, the sample was seeded into Matrigel in 24-well plates. Following Matrigel polymerisation (10 min at 37 °C), 500 μl human culture media (Advanced DMEM/F12 containing, 1× N2 (Life Technologies), 10 mM HEPES, 2 mM GlutaMAX, 1× B27, 10 nM gastrin I (Biogems), 50 ng/ml recombinant EGF, 500 nM A83-01 (Biogems), 100 ng/ml recombinant Noggin (Peprotech), 500 ng/ml R-spondin-1 (Peprotech), 10 μM SB202190 (Sigma) 10 μM Y-27632 (Abmole), 10 mM nicotinamide (Sigma), 1 mM *N*-acetylcysteine (Sigma) and penicillin/streptomycin. The sample was transduced with 500 μl of virus supernatant (scrambled or CSN6-specific shRNA) in the presence of polybrene (Millipore, TR-1003-G) and incubated for 8 h, then changed the fresh culture medium and renewed the culture medium every 3 days. The organoid numbers in each well were quantified at day 12.

### Patients and tissue samples

For analysis, the mRNA expression level of *Aldh1a1* and *Csn6* in CRC was quantified. Thirteen paired CRC and normal colon specimens were collected from the Department of Surgery at the Sixth Affiliated Hospital of Sun Yat-sen University.

For tissue microarray (TMA), we obtained paraffin-embedded samples of primary colorectal adenocarcinomas from CRC patients. Total of 267 samples were collected from the First Affiliated Hospital of Sun Yat-sen University with the patients’ written informed consent and approval from study centre’s Institutional Review Board. The immunostained slides were scanned by Aperio Versa (Leica Biosystems). The total 267 samples were classified into four groups based on the expression levels of CSN6 and TRIM21. The receiver operating characteristic (ROC) curve was used to define the “High expression” and “Low expression” by the SPSS software.

### Data mining

#### Correlation

The Cancer Genome Atlas (TCGA) for 255 colorectal adenocarcinoma patients’ tumour samples and 238 patients from GSE17538 were downloaded from the publicly available Gene Expression Omnibus (GEO) databases. The correlation of *Csn6* and *Aldh1a1* was performed by the GraphPad Prism.

#### Survival analysis

Five hundred and eighty-five patients from GSE39582 were downloaded from the publicly available GEO databases and combined with the patients’ clinical information. Samples with missing relapse-free survival status/time were removed from the analysis. The ROC curve was generated to separate the samples based on the gene expression level (*Csn6* high expression and *Csn6* low expression, *Aldh1a1* high expression and *Aldh1a1* low expression). Combining the *Csn6* expression and *Aldh1a1* expression together, the samples were separated into four groups (*Csn6* low and *Aldh1a1* low (*n* = 109), *Csn6* low and *Aldh1a1* high (*n* = 286), *Csn6* high and *Aldh1a1* low (*n* = 59) and *Csn6* high and *Aldh1a1* high (*n* = 120)). A relapse survival curve was generated by Kaplan–Meier.

### Knockout (KO) cell lines

TRIM21 KO was performed as previously described.^29^ In HEK293T and HCT116 cells, target sequences were cloned into pSpCas9-2A-GFP(PX458) by cutting with BbsI, then the cells were transfected with the plasmid containing TRIM21 sgRNA. Forty-eight hours after transfection, the cells with green fluorescent protein (GFP) were sorted by flow cytometry, the GFP-positive cells were then diluted and expanded to get single clones. Western blotting was used to identify TRIM21 KO cells.

### Immunoprecipitation and western blotting

Cells were transfected with the indicated plasmids. Forty-eight hours after transfection, the cells were treated with MG132 for 6 h and lysed in lysis buffer (50 mM Tris-Cl, pH 7.5, 0.1% NP-40, 0.1%Triton-100,150 mM NaCl, 0.1 M EDTA, a cocktail of phosphate and proteinase inhibitors (bimake)) for 30 min at 4 °C. For immunoprecipitations, the cell lysates were incubated with Anti-Flag M2 Magnetic Beads (Sigma) overnight at 4 °C. The beads were boiled 5 min after extensive washing. Protein samples were separated by sodium dodecyl sulfate–polyacrylamide gel electrophoresis (SDS–PAGE).

For endogenous co-immunoprecipitation (Co-IP), HEK293T cells were treated with MG132 for 6 h before they were collected, and the cell lysates were prepared as described above. For immunoprecipitations, TRIM21 antibody or the control IgG antibody were added into cell lysates and incubated overnight at 4 °C, then 50 μl of either Protein A or Protein G beads (Santa Cruz Biotechnology) for 2 h. The beads were washed for four times with lysis buffer and boiled with 2× loading buffer for 5 min. Protein samples were separated by SDS–PAGE.

For western blot analysis, the cells were washed with PBS and lysed in lysis buffer, then sonicated and centrifugated and the supernatant was boiled with 1× loading buffer for 5 min. Protein samples were separated by SDS–PAGE. We used antibodies against Flag-Tag (Cell Signaling, #8146), HA-Tag (Cell Signaling, #3724), Myc-Tag (Cell Signaling, #2278), CSN6 (Enzo, BML-PW8295), TRIM21 (abcam, ab119859), OCT1 (Cell Signaling, #8157) and ALDH1A1 (Cell Signaling, #54135).

### Turnover assay

Cells were transfected with the indicated plasmids. For viral transfection, cells were first transfected with the indicated virus and were then seeded into 6-well plates. Twenty-four hours later, cells were treated with 60 μg/ml cycloheximide and harvested at the indicated times after cycloheximide treatment. The protein levels were analysed by western blotting.

### Ubiquitination assay

HEK293T cells were co-transfected with the indicated plasmids for 48 h. Then cells were treated with 50 μg/ml MG132 for 6 h. Cell lysates were pulled down with anti-Flag or Ni-NTA agarose. The beads were boiled with 2× loading buffer for 5 min, after extensive washing. The protein levels were analysed by western blotting with the indicated antibodies.

### Immunohistochemistry (IHC)

Tumour tissues were fixed in 4% paraformaldehyde and were then embedded in paraffin by a company (Servicebio). Paraffin-embedded slides were deparaffinised in xylene and rehydrated in a graded ethanol series. Slides were processed for antigen retrieval by microwave heating for 15 min in 1× EDTA unmasking solution (Origene), cooled for 30 min and incubated in 3% hydrogen peroxide for 10 min. After blocking with blocking solution (Origene) for 1 h at room temperature, slides were incubated in diluted primary antibody overnight at 4 °C. The next day, after incubation with biotinylated goat anti-rabbit or anti-mouse IgG at room temperature for 30 min, immunostaining was visualised with diaminobenzidine, and sections were then counterstained with haematoxylin. Antibodies against CSN6 (Enzo, BML-PW8295), TRIM21 (Abcam, ab119859), OCT1 (Cell Signaling, #8157) and ALDH1A1 (Cell Signaling, #54135) were used.

### Statistical analysis

SPSS software was used for survival analysis, and Kaplan–Meier analyses were used to generate survival curves. Log-rank test was used to calculate *P* values. The data are presented as the mean ± sd. Differences between the two groups were calculated by unpaired Student’s *t* tests. One-way analysis of variance followed by Bonferroni test was performed for comparisons among multiple groups.

## Results

### CSN6 expression instigates stemness through ALDH1A1

**Fig. 1:**
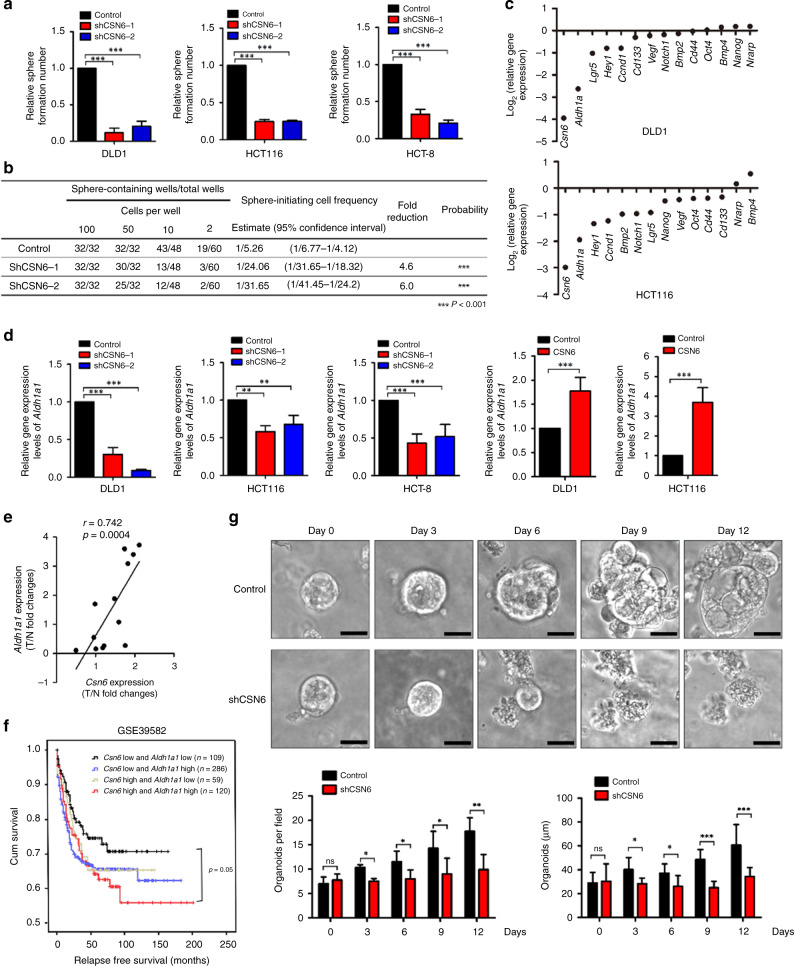
CSN6 is required for sphere formation and initiates stemness through ALDH1A1. **a** Sphere-formation assay of DLD-1, HCT116 and HCT-8 cells carrying scrambled or CSN6-specific shRNA. **b** DLD-1 cells carrying scrambled or CSN6-specific shRNA were dissociated into a single-cell suspension, seeded in 96-well plates with an ultra-low attachment surface at a density of 2, 10, 50 or 100 cells per well and cultured for 12 days. The frequency of sphere-initiating cells was estimated using the ELDA software. **c** Quantitative RT-PCR analysis was performed to measure the mRNA levels of stem cell markers (*Aldh1a1*, *Lgr5*, *Cd133* and *Cd44*), embryonic stem cell components (*Nanog* and *Oct4*), WNT pathway components (*Vegf* and *Ccnd1*), Notch pathway components (*Notch1*, *Hey1* and *Nrarp*) and BMP family genes (*Bmp2* and *Bmp4*) in DLD-1 cells and HCT116 cells carrying scrambled or CSN6-specific shRNA. **d** Quantitative RT-PCR analysis was performed to measure the mRNA levels of *Aldh1a1* in DLD-1, HCT116 and HCT-8 cells with CSN6 knockdown or CSN6 overexpression. **e** Quantitative RT-PCR analysis was performed to measure the mRNA levels of colorectal cancer and adjacent colorectal tissues. The levels of *Csn6* were positively correlated with the expression of *Aldh1a1* at mRNA levels in 13 pairs of human colorectal carcinomas (T) with matched normal tissues (N). **f** Kaplan–Meier survival curves of relapse-free survival time based on *Csn6* and *Aldh1a1* expression in CRC tissues. *P < 0.05, **P < 0.01 and ***P < 0.001. **g** Knockdown of CSN6 affected patient-derived tumour organoid (tumour PDO) growth. The morphology of the organoids is shown. The number of organoids growing to a size of >25 μm was calculated. Scale bars, 25 μm.

It has been suggested that CRC could be driven by a small population of CSCs, which are self-renewing and multipotent, and that stem cells could be responsible for the recurrence of CRC. Cancer cells are able to form three-dimensional multicellular spheroids under nonadherent culture conditions. Cell populations that can form spheres are enriched in CSCs. To investigate the role of CSN6 in affecting CRC CSCs, we first knocked down CSN6 in the human colon cancer cell lines HCT116, DLD-1 and HCT-8 via CSN6-targeted shRNA or control shRNA and then performed sphere-formation assays. Knockdown of CSN6 reduced the number of spheres formed in the HCT116, DLD-1 and HCT-8 cell lines (Fig. 1a and S1A). Consistent with this finding, CSN6 knockdown decreased the sphere-initiation frequency, as determined by in vitro limited dilution assays (LDAs; Fig. 1b). Furthermore, migration/invasion assays showed that knockdown of CSN6 reduced cell migration and invasion in DLD-1 (Fig. S1B). These results suggest that CSN6 plays an important role in regulating CRC stemness.

Intestinal stem cell markers (*Aldh1a1*, *Lgr5*, *Cd133*, *Cd44*) play a critical role in CRC.^30^ Transcription factors, including *Oct4* and *Nanog*, can regulate embryonic stem cell pluripotency.^31^ We sought to examine the impact of CSN6 on the expression of these genes that may facilitate sphere formation. Knockdown of CSN6 in the DLD-1 cell line led to reduced mRNA levels of the stem cell marker *Aldh1a1*, as measured by qPCR (Fig. 1c). qPCR analysis revealed that the mRNA levels of the stem cell marker *Aldh1a1* were decreased more than those of other stem cell markers (*Lgr5*, *Cd133*, *Cd44*), WNT target genes (*Vegf* and *Ccnd1*) and Notch signalling-related genes (Fig. 1c); therefore, we further focused on *Aldh1a1*.

Accordingly, CSN6 knockdown led to a decrease in the level of *Aldh1a1* mRNA (Fig. 1d), while ectopic expression of CSN6 in both the HCT116 and DLD-1 cell lines resulted in increased *Aldh1a1* mRNA levels (Fig. 1d). Furthermore, *Csn6* expression also showed a significant positive correlation with *Aldh1a1* expression in two sets of colon cancer data, the TCGA database and GSE17538 (Fig. S1C). To determine the clinical relevance, we examined the gene expression levels of *Csn6* and *Aldh1a1* in patient CRC tissues. Tissues with high *Csn6* expression had high expression of *Aldh1a1* (Fig. 1e). Kaplan–Meier analysis showed that high levels of both *Csn6* and *Aldh1a1* correlated with poor relapse-free survival in the colon cancer GSE39582 data set (Fig. 1f). We also generated PDOs^32^ from CRC patient specimens. Knockdown of CSN6 reduced the number and the size of CRC PDOs formed (Fig. 1g). A key implication of the above data is that CSN6 exerts cancer stemness-initiating activity and is critical in maintaining spheroid formation and PDO proliferation. Collectively, these results support the idea that CSN6 regulates CRC stemness by controlling ALDH1A1.

### CSN6 regulates ALDH1A1 expression through TRIM21

To investigate how knockdown of CSN6 can lead to reduced expression of *Aldh1a1* mRNA, we determined whether CSN6-associated proteins might have a role. We performed mass spectrometry to identify CSN6-associated proteins, and we found that the RING domain-containing ubiquitin E3 ligase TRIM21 is one of the associated proteins (data not shown). A previous study showed that TRIM21 could control the stability of OCT1,^33^ a transcriptional activator of *Aldh1a1*. As CSN6 is involved in regulating protein stability, especially impacting the RING domain-containing E3 ubiquitin ligases,^16,17^ we hypothesised that CSN6 regulates ALDH1A1 expression by controlling the TRIM21–OCT1 axis.

**Fig. 2:**
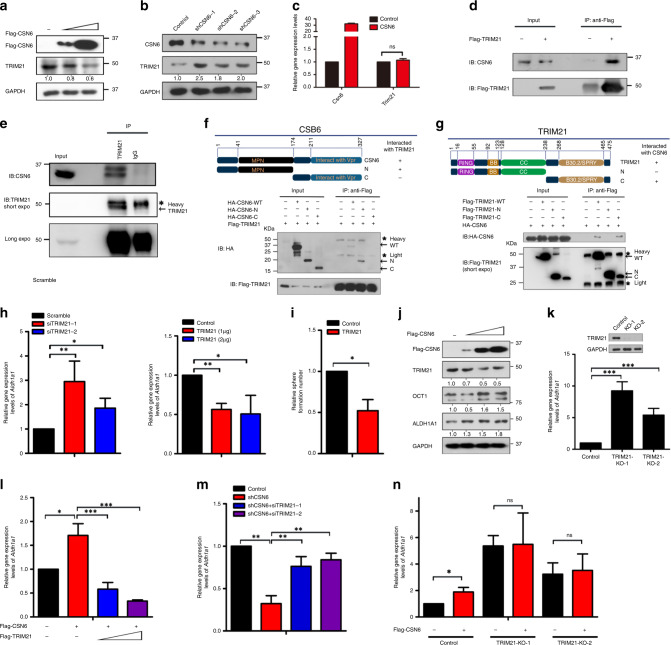
CSN6 interacts with the TRIM21 E3 ligase and regulates ALDH1A1 through regulating TRIM21. **a** DLD-1 cells were transfected with Flag-CSN6 plasmids. The protein level of TRIM21 was immunoblotted with anti-TRIM21 antibodies. **b** DLD-1 cells were infected with scrambled or CSN6-specific shRNA lentivirus. The protein level of TRIM21 was immunoblotted with anti-TRIM21 antibodies. **c** Quantitative RT-PCR analysis was performed to measure the mRNA levels of *Trim21* in cells transfected with empty vector or Flag-CSN6. **d** Flag-TRIM21 was expressed in HCT116 cells. Flag-TRIM21 was immunoprecipitated with anti-Flag, and the associated CSN6 was detected by western blotting. **e** HEK293T cell lysates were immunoprecipitated with an anti-TRIM21 antibody and immunoblotted with anti-TRIM21 and anti-CSN6 antibodies. **f** HEK293T cells were co-transfected with Flag-TRIM21 and the HA-CSN6-WT, CSN6-N terminal or CSN6-C terminal construct. Cell lysates were immunoprecipitated with anti-Flag and subsequently immunoblotted with anti-Flag and anti-HA antibodies. Heavy heavy immunoglobulin chain, Light light immunoglobulin chain. **g** HEK293T cells were co-transfected with HA-CSN6 and the Flag-TRIM21-WT, TRIM21-N terminal or TRIM21-C terminal construct. Cell lysates were immunoprecipitated with anti-Flag and subsequently immunoblotted with anti-Flag and anti-HA antibodies. **h** Quantitative RT-PCR analysis was performed to measure the mRNA levels of *Aldh1a1* in HCT116 cells carrying scrambled or TRIM21-specific siRNA. Quantitative RT-PCR analysis was performed to measure the mRNA levels of *Aldh1a1* in HCT116 cells carrying empty vector or Flag-TRIM21 plasmids. **i** Sphere-formation assay of DLD-1 cells transduced with control or Flag-TRIM21 lentivirus. **j** HCT116 cells were transfected with Flag-CSN6 plasmids. The protein level of TRIM21, OCT1 and ALDH1A1 was immunoblotted with anti-TRIM21, anti-OCT1 and anti-ALDH1A1 antibodies. **k** Quantitative RT-PCR analysis was performed to measure the mRNA levels of *Aldh1a1* in HCT116 cells with TRIM21 knockout. **l** Quantitative RT-PCR analysis was performed to measure the mRNA levels of *Aldh1a1* in HCT116 cells stably expressing empty vector or Flag-CSN6 and rescued with Flag-TRIM21 plasmids. **m** Quantitative RT-PCR analysis was performed to measure the mRNA levels of *Aldh1a1* in HCT116 cells stably expressing scrambled or CSN6-specific shRNA and rescued with TRIM21-specific siRNA. **n** The knockout efficiency of TRIM21 in HCT116 cells by TRIM21 sgRNAs was evaluated by western blotting. Quantitative RT-PCR analysis was performed to measure the mRNA levels of *Aldh1a1* in control or HCT116 cells with TRIM21 knockout and then transfected with empty vector or Flag-CSN6. *P < 0.05, **P < 0.01 and ***P < 0.001.

We first identified that overexpression of CSN6 reduced the steady-state expression of TRIM21 in a dose-dependent manner in the DLD-1 cell line (Fig. 2a). In addition, knockdown of CSN6 increased TRIM21 protein levels (Fig. 2b). However, CSN6 overexpression did not alter the mRNA expression of TRIM21 (Fig. 2c), suggesting that CSN6 can regulate TRIM21 at the posttranscriptional level. Then we confirmed that CSN6 could interact with TRIM21 in HCT116 cells by Co-IP (Fig. 2d). Endogenous interaction was also demonstrated (Fig. 2e). We divided the CSN6 into CSN6-N terminal (1–174 aa) and CSN6-C terminal (175–327 aa). The MPN domain (41–174 aa) is located in N-terminal and is indispensable in CSN6’s impact on Cullin-1 neddylation.^17^ Then we further identified the domain of CSN6 responsible for its interaction with TRIM21 and found that the MPN domain of CSN6 bound to TRIM21 (Fig. 2f). Members of the TRIM family have three conserved domains: the RING, B-box, coiled-coil domain, and a B30.2 (or PRYSPRY) region. We found that the TRIM21 B30.2/SPRY domain is involved in binding CSN6 (Fig. 2g), consistent with the observation that the B30.2/SPRY domain is responsible for mediating protein–protein interactions.

Quantitative reverse transcription PCR (RT-PCR) analysis demonstrated an increase in the *Aldh1a1* mRNA level in cells with TRIM21 knockdown, and overexpression of TRIM21 led to a decrease in the *Aldh1a1* mRNA level (Fig. 2h). Owing to TRIM21’s negative effect on the expression of *Aldh1a1*, TRIM21 expression can reduce the sphere-formation ability of CRC cells (Fig. 2i). Next, we investigated whether CSN6 regulates the ALDH1A1 expression through the TRIM21–OCT1 axis. We found that the expression of CSN6 increased the steady-state protein expression levels of OCT1 and ALDH1A1, with a concurrent reduction in TRIM21 expression in a dose-dependent manner (Fig. 2j). Again, an increase in the *Aldh1a1* mRNA level was observed in TRIM21 KO cells (Fig. 2k). Importantly, CSN6 caused an increase in the *Aldh1a1* mRNA level and that overexpression of TRIM21 led to a decrease in the *Aldh1a1* mRNA level, even in the presence of CSN6 (Fig. 2l). In addition, quantitative RT-PCR analysis indicated that CSN6 knockdown caused a decrease in the *Aldh1a1* mRNA level but failed to do so when TRIM21 was also knocked down (Fig. 2m). We further showed that CSN6 expression caused a significant increase in the *Aldh1a1* mRNA level in control cells but not in TRIM21 KO cells (Fig. 2n). Collectively, the above results consistently suggest that CSN6 interacts with TRIM21, which in turn impacts *Aldh1a1* mRNA expression.

### CSN6 promotes ubiquitination-mediated degradation of the TRIM21 E3 ubiquitin ligase

**Fig. 3:**
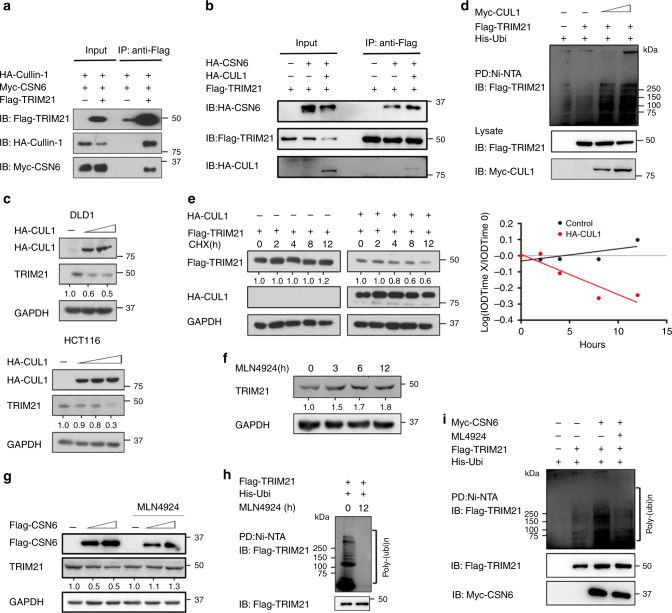
CSN6 and CUL1 affect the steady-state expression, ubiquitination level and turnover rate of TRIM21. **a** HEK293T cells were co-transfected with Flag-TRIM21, HA-CUL1 and Myc-CSN6. Flag-TRIM21 was immunoprecipitated with anti-Flag, and the associated Cullin1 and CSN6 were detected by western blotting with anti-Flag, anti-HA and anti-Myc antibodies. **b** HEK293T cells were co-transfected with the indicated plasmids. Flag-TRIM21 was immunoprecipitated with anti-Flag, and the associated Cullin1 and CSN6 were detected by western blotting with anti-Flag and anti-HA antibodies. **c** DLD-1 and HCT116 cells were transfected with HA-CUL1 plasmids. The protein levels of TRIM21 were immunoblotted with anti-TRIM21 antibodies. **d** HEK293T cells were co-transfected with His-Ubi, Flag-TRIM21 and Myc-CUL1. MG132 was added to the cells 6 h before they were harvested. Cell lysates were pulled down with Ni-NTA beads and immunoblotted with anti-Flag and anti-Myc antibodies. **e** HEK293T cells were transfected with Flag-TRIM21 and HA-CUL1. Overexpression of CUL1 increased the turnover rate of the TRIM21 protein. CHX cycloheximide, IOD integrated optical density. **f** HCT116 cells were treated with MLN4924 (3 μM) at the indicated time points, and cell lysates were immunoblotted with the indicated antibodies. **g** HCT116 cells were transfected with Flag-CSN6 plasmids, and MLN4924 (3 μM) was added to the cells 24 h before they were harvested. The protein levels of TRIM21 were immunoblotted with anti-TRIM21 antibodies. **h** HEK293T cells were co-transfected with His-Ubi and Flag-TRIM21, and MLN4924 (3 μM) was then added to the cells at the indicated time points. MG132 was added to the cells 6 h before they were harvested. Cell lysates were pulled down (PD) with Ni-NTA beads and the ubiquitination of TRIM21 was immunoblotted with anti-Flag antibodies. **i** HEK293T cells were co-transfected with His-Ubi, Flag-TRIM21 and Myc-CSN6, and MLN4924 (3 μM) was then added to the cells 24 h before they were harvested. MG132 was added to the cells 6 h before they were harvested. Cell lysates were pulled down with Ni-NTA beads and immunoblotted with anti-Flag and anti-Myc antibodies.

TRIM21 can interact with SKP2 and Cullin1 and form a TRIM21-containing SCF (SKP2)-like complex to promote ubiquitination of its substrates.^34^ Furthermore, neddylation of Cullin proteins is important to Cullin-RING ubiquitin ligase activation.^35^ Interestingly, CSN6 can increase CUL1 neddylation.^17^ We hypothesised that CSN6 might regulate TRIM21 ubiquitination by increasing CUL1 neddylation. To verify Cullin’s involvement, we first showed that TRIM21 interacted with CUL1 and CSN6 by Co-IP (Fig. 3a). TRIM21 binding with CSN6 is enhanced by the presence of CUL1 (Fig. 3b). CUL1 can affect the steady-state expression level, ubiquitination level and turnover rate of TRIM21 (Fig. 3c–e). To further investigate whether neddylation of CUL1 is critical in regulating TRIM21 ubiquitination, we treated HCT116 cells with a neddylation inhibitor (MLN4924)^36^ at different time points. Western blotting showed that MLN4924 treatment increased the TRIM21 protein level (Fig. 3f). In HCT116 cells, CSN6 reduced the steady-state level of TRIM21, and MLN4924 treatment reversed this effect (Fig. 3g). Ubiquitination assays showed that MLN4924 treatment reduced TRIM21 ubiquitination (Fig. 3h). Moreover, MLN4924 treatment abrogated TRIM21 ubiquitination promoted by CSN6 (Fig. 3i). These findings highlight the complexity of TRIM21 regulation and indicate that CSN6-CUL1 regulation is involved in TRIM21 ubiquitination.

**Fig. 4:**
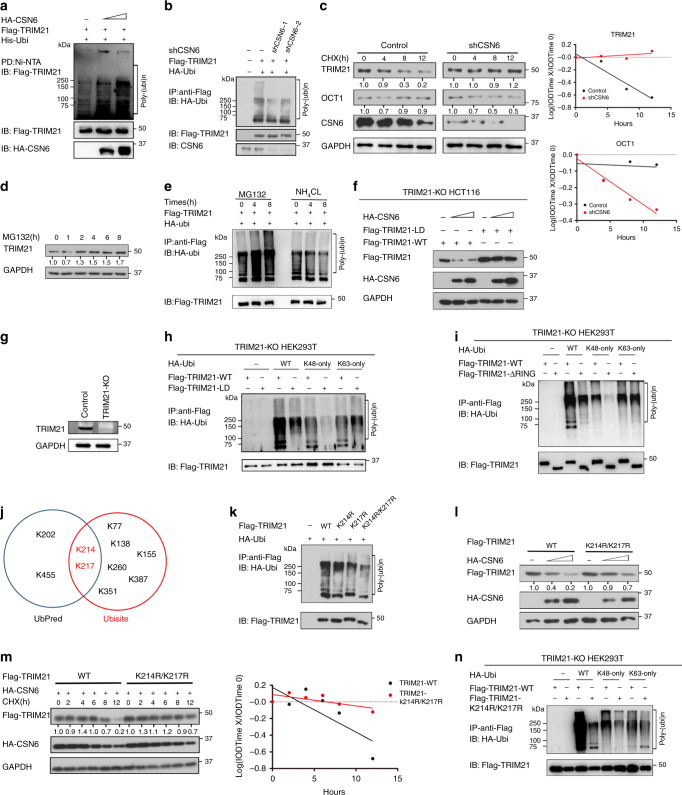
CSN6 facilitates ubiquitination-mediated degradation of TRIM21. **a** HEK293T cells were transfected with His-Ubi together with Flag-TRIM21 and HA-CSN6. MG132 was added to the cells 6 h before they were harvested. Cell lysates were pulled down with Ni-NTA beads and immunoblotted with anti-Flag and anti-HA antibodies. **b** HEK293T cells were transfected with HA-Ubi and Flag-TRIM21 and subsequently treated with shCSN6. MG132 was added to the cells 6 h before they were harvested. Cell lysates were pulled down with anti-Flag and immunoblotted with the anti-Flag and anti-HA antibodies. **c** In DLD-1 cells expressing scrambled or CSN6-specific shRNA, CSN6 knockdown reduced the turnover rate of the TRIM21 protein and increased the turnover rate of OCT1. CHX cycloheximide. **d** DLD-1 cells were seeded into six-well plates. Forty-eight hours later, the cells were treated with MG132 at the indicated time points. **e** HEK293T cells were co-transfected with HA-Ubi and Flag-TRIM21. Forty-eight hours later, MG132 or NH_4_Cl was added to the cells at the indicated time points. Cell lysates were pulled down with anti-Flag and immunoblotted with anti-Flag and anti-HA antibodies. **f** HCT116 cells were co-transfected with HA-CSN6 and Flag-TRIM21-WT or Flag-TRIM21-LD (C16A, C31A and H33W) mutant. Cell lysates were immunoblotted with anti-Flag and anti-HA antibodies. **g** The knockout efficiency of TRIM21 in HEK293T cells by TRIM21 sgRNAs was evaluated by western blotting. **h** HEK293T cells were co-transfected with the HA-Ubi-WT, Ubi-K48 only or Ubi-K63 only construct and the Flag-TRIM21-WT or Flag-TRIM21-LD construct. MG132 was added to the cells 6 h before they were harvested. Cell lysates were pulled down with anti-Flag and immunoblotted with anti-Flag and anti-HA antibodies. **i** HEK293T KO cells were co-transfected with the indicated HA-Ubi constructs and Flag-TRIM21 or the Flag-TRIM21 ΔRING mutant. MG132 was added to the cells 6 h before they were harvested. Cell lysates were pulled down with anti-Flag and immunoblotted with anti-HA antibodies. **j** Prediction of TRIM21 ubiquitination sites by UbiSite (http://140.138.144.145/~ubinet/index.php) and UbPred (http://www.ubpred.org/index.html). **k** HEK293T cells were co-transfected with HA-Ubi and Flag-TRIM21-WT or its mutants. MG132 was added to the cells 6 h before they were harvested. Cell lysates were pulled down with anti-Flag and immunoblotted with anti-Flag and anti-HA antibodies. **l** HCT116 cells were co-transfected with HA-CSN6 and Flag-TRIM21-WT or the Flag-TRIM21 K214R/K217R mutant. Cell lysates were immunoblotted with anti-Flag and anti-HA antibodies. **m** HCT116 cells were co-transfected with HA-CSN6 and Flag-TRIM21 or the Flag-TRIM21 K214R/K217R mutant and were then treated with cycloheximide. Cell lysates were immunoblotted with anti-Flag and anti-HA antibodies. **n** HEK293T KO cells were co-transfected with the indicated HA-Ubi constructs and Flag-TRIM21-WT or the Flag-TRIM21 K214R/K217R mutant. MG132 was added to the cells 6 h before they were harvested. Cell lysates were pulled down with anti-Flag and immunoblotted with anti-HA antibodies.

To further examine the role of CSN6 in regulating TRIM21 ubiquitination, we showed that overexpression of CSN6 increases the ubiquitination level of TRIM21 (Fig. 4a). Consistent with this finding, knockdown of CSN6 reduces the ubiquitination level of TRIM21 and decelerated turnover of TRIM21 (Fig. 4b, c). Treatment with the proteasome inhibitor MG132 led to increased TRIM21 protein levels (Fig. 4d). Furthermore, the results of the ubiquitination assay showed that treatment with MG132 but not treatment with the lysosome inhibitor ammonium chloride (NH_4_Cl)^37^ increased the TRIM21 ubiquitination level (Fig. 4e), indicating that TRIM21 degradation depends on the proteasome rather than on lysosomes. Importantly, CSN6 reduced the steady-state expression of TRIM21-WT but not a TRIM21 (C16A, C31A and H33W) RING-finger ((ligase-dead (LD)) mutant in a dose-dependent manner in HCT116 cells with TRIM21 KO (Fig. 4f). We further showed that exogenous TRIM21 LD had a reduced level of ubiquitination compared to that of wild-type TRIM21 in TRIM21 KO HEK293T cells (Fig. 4g, h), suggesting that mutation of the TRIM21 RING-finger domain is responsible for this decrease. This finding implies that TRIM21 is partly ubiquitinated through its ligase domain/RING finger domain. This ligase domain/RING finger domain–mediated ubiquitination occurs through K48 linkage, as K48-only ubiquitin cannot be utilised by the TRIM21 LD mutant (Fig. 4h). Notably, the TRIM21 LD mutant was still ubiquitinated in the presence of K63-only ubiquitin, suggesting that other E3 ligases may mediate K63-linked ubiquitination of TRIM21. We obtained a similar result in a similar ubiquitination assay in which TRIM21 RING domain deletion (TRIM21-ΔRING) replaced TRIM21 LD mutant (Fig. 4i). Together, these results indicate that CSN6-promoted TRIM21 degradation is dependent on TRIM21 self-ubiquitination via K48 ubiquitin linkage.

We identified the potential ubiquitination residues on TRIM21 by analysing the TRIM21 sequence using the web tools UbiNet (http://140.138.144.145/~ubinet/index.php) and UbPred (http://www.ubpred.org/index.html) and found that the K214 and K217 residues were predicted by both UbiNet and UbPred as the ubiquitination residues on TRIM21 (Fig. 4j). Indeed, replacing these residues with Arg (K214R or K217R) markedly reduced TRIM21 ubiquitination, and the double mutation (K214R/K217R) further reduced TRIM21 ubiquitination (Fig. 4k). Consistent with these findings, CSN6’s impact on decreasing the steady-state expression of the TRIM21 K214R/K217R mutant was compromised (Fig. 4l). Furthermore, these K to R mutants also exhibited a reduced turnover rate (Fig. 4m). Ubiquitination assays revealed that K214 and K217 are the major sites that can be ubiquitinated through K48 linkage (Fig. 4n).

**Fig. 5:**
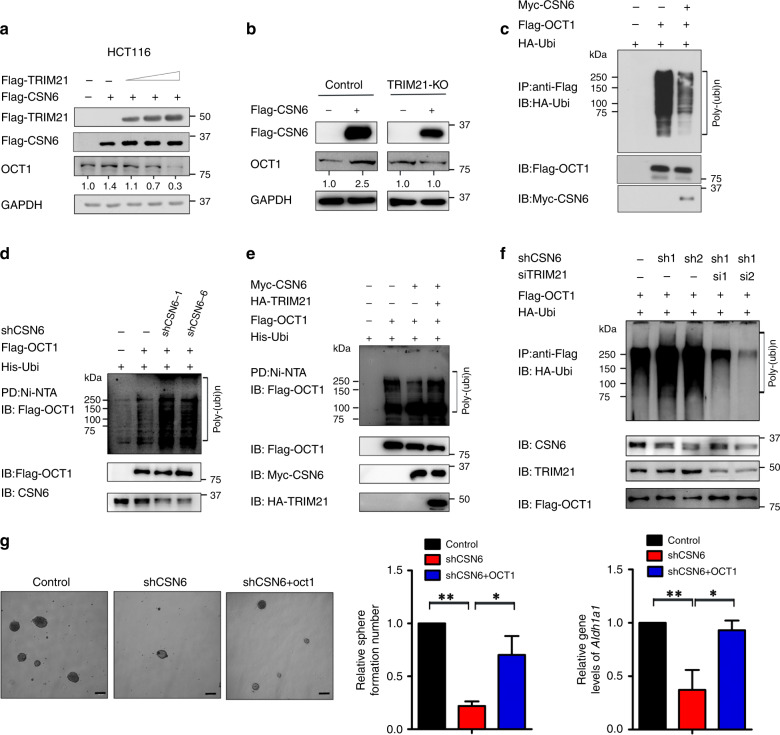
The CSN6–TRIM21–OCT1 axis is involved in ALDH1A1 expression. **a** HCT116 cells were transfected with the indicated plasmids. Cell lysates were immunoblotted with anti-Flag and anti-OCT1 antibodies. **b** TRIM21 knockout cells were transfected with the indicated plasmid, and cell lysates were immunoblotted with anti-Flag and anti-OCT1 antibodies. **c** HEK293T cells were transfected with HA-Ubi and the indicated plasmids. Cell lysates were subjected to IP with anti-Flag and immunoblotted with anti-HA antibodies. **d** HEK293T cells were transfected with His-Ubi and the indicated plasmids. Cell lysates were subjected to PD with Ni-NTA beads and immunoblotted with anti-Flag and anti-CSN6 antibodies. **e** HEK293T cells were transfected with the indicated plasmids. Cell lysates were subjected to PD with Ni-NTA beads and immunoblotted with anti-Flag antibodies. **f** HEK293T cells were transfected with HA-Ubi and Flag-OCT1 and subsequently treated with shCSN6 and siTRIM21. MG132 was added to the cells 6 h before they were harvested. Cell lysates were pulled down with anti-Flag and immunoblotted with anti-Flag and anti-HA antibodies. **g** DLD-1 cells were engineered to stably express scrambled or CSN6-specific shRNA and were then rescued with Flag-OCT1 plasmids. Sphere formation was measured. Quantitative RT-PCR analysis was performed to measure the mRNA levels of *Aldh1a1*. *P < 0.05 and **P < 0.01.

TRIM21 is the ubiquitin E3 ligase for OCT1, a transcriptional activator of ALDH1A1^33^. We sought to determine whether CSN6’s negative effect on TRIM21 translates to its impact on OCT1. Indeed, CSN6 knockdown led to decelerated turnover of TRIM21 but accelerated turnover of OCT1 (Fig. 4c). CSN6’s positive impact on OCT1 was reversed by overexpression of TRIM21 (Fig. 5a). In addition, CSN6’s effect on increasing the steady-state expression of OCT1 was compromised in TRIM21 KO cells (Fig. 5b). We found that overexpression of CSN6 reduced the ubiquitination level of OCT1 (Fig. 5c). Consistent with this finding, we found that knockdown of CSN6 increased the ubiquitination level of OCT1 (Fig. 5d). Moreover, overexpression of CSN6 reduced OCT1 ubiquitination, while ectopic expression of TRIM21 reversed this effect (Fig. 5e). Simultaneously, knockdown of CSN6 increased OCT1 ubiquitination, while knockdown of TRIM21 reversed this impact (Fig. 5f). In terms of stemness regulation, CSN6 knockdown reduced the sphere-formation ability (Fig. 5g), while overexpression of OCT1 in the presence of CSN6 knockdown rescued the sphere-formation ability (Fig. 5g). Quantitative RT-PCR analysis showed a significant decrease in the *Aldh1a1* mRNA level in cells with CSN6 knockdown, and overexpression of OCT1 in the presence of CSN6 knockdown restored the expression level of *Aldh1a1* mRNA (Fig. 5g). Together, these data establish the CSN6–TRIM21–OCT1 axis as a regulator of the *Aldh1a1* mRNA level and demonstrate the impact of this axis on stemness regulation.

### CSN6 regulates the TRIM21–OCT1–AlDH1A1 axis to promote cancer stemness during tumorigenesis

**Fig. 6:**
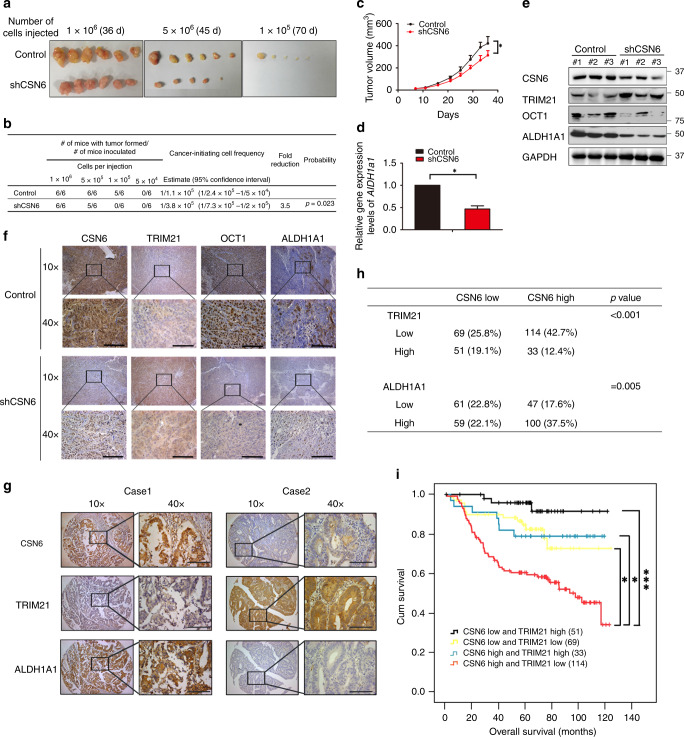
CSN6-mediated regulation of CRC stemness via the TRIM21–OCT1–AlDH1A1 axis is involved in tumorigenesis. **a** Frequency of CSCs in DLD-1 cells transduced with control non-silencing shRNA (shCtrl) or shCSN6 lentivirus, as measured by an LDA in vivo. Tumour morphology in each group. **b** Quantification of tumour initiation. **c** Tumour growth curve for mice subcutaneously injected with control or shCSN6 DLD-1 cells (1 × 10^6^ cells per mouse, six mice per group). **d** RNA was extracted from tumour tissues. Quantitative RT-PCR analysis was performed to measure the mRNA level of *Aldh1a1*. The data are presented as the means ± SDs. **e** Protein was extracted from the tumour tissues described in **c** and immunoblotted with the indicated antibodies. **f** Representative IHC staining for CSN6, TRIM21, OCT1 and ALDH1A1 in tumour tissues from mice. The scale bars represent 100 μm. **g** Representative IHC staining for CSN6, TRIM21 and ALDH1A1 in human CRC TMAs. Case 1 is representative of a patient with CSN6-high colon cancer. Case 2 is representative of a patient with non-CSN6-high colon cancer. The scale bars represent 100 μm. **h** Quantification of staining intensities from sections in **g**. CSN6 and TRIM21 show a negative correlation, while CSN6 and ALDH1A1 show a positive correlation. **i** Kaplan–Meier survival curves of overall survival time based on CSN6 and TRIM21 expression from TMA analysis. *P < 0.05 and ***P < 0.001.

To investigate the role of the CSN6–TRIM21–OCT1–ALDH1A1 axis in promoting colon cancer tumour stemness, we performed in vivo LDAs to monitor tumour initiation.^38^ A limited dilution series of DLD-1 cells expressing CSN6-targeted shRNA or control shRNA were injected into athymic nude mice, and tumour incidence was monitored over 2 months. Knockdown of CSN6 led to reduction of tumour-formation frequency in vivo at low diluted level (Fig. 6a, b). Low numbers (10^5^ cells) of CSN6 knockdown cells were not tumorigenic in immunodeficient mice, while control cells were still tumorigenic in these low numbers (Fig. 6a, b). We also found that knockdown of CSN6 reduced tumour growth (Fig. 6c). Quantitative RT-PCR analysis showed that the mRNA level of *Aldh1a1* was decreased in shCSN6-expressing xenograft tumour samples (Fig. 6d). Consistent with this finding, the results of western blotting and IHC staining revealed that the expression of TRIM21 was increased but the expression of OCT1 and ALDH1A1 was reduced in shCSN6-expressing xenograft tumour samples (Fig. 6e, f). The results of these in vivo functional analyses via animal experiments clearly recapitulate our biochemical observations and support the idea that CSN6 is a regulator of cancer stemness.

To examine and confirm the relationship among CSN6, TRIM21 and ALDH1A1 in human cancers, we performed IHC staining on a TMA from our human colon cancer cohort to assess the expression of CSN6, TRIM21 and ALDH1A1 (Fig. 6g and Table S2). In CRC tumour tissue, CSN6 and TRIM21 showed a significant negative correlation in staining intensity (Fig. 6h), while CSN6 and ALDH1A1 showed a significant positive correlation in staining intensity (Fig. 6h). On the basis of the expression levels of CSN6 and TRIM21, the samples were classified into four groups: High CSN6 and Low TRIM21 expression, High CSN6 and High TRIM21 expression, Low CSN6 and Low TRIM21 expression, and Low CSN6 and High TRIM21 expression. Kaplan–Meier analysis results indicated that, compared with the other groups, patients with High CSN6 and Low TRIM21 expression (114 patients) tended to exhibit the poorest overall survival (Fig. 6i). Together, our data demonstrate that CSN6-facilitated self-ubiquitination and subsequent degradation of TRIM21 alters cancer stemness and that overexpression of CSN6 in cancer leads to deregulation of the TRIM21–OCT1–ALDH1A1 axis, thereby promoting cancer stemness during tumorigenesis (Fig. S2).

## Discussion

CSN6 is involved in a wide range of regulatory processes, including cell cycle control, signal transduction, metabolism and tumorigenesis. Here we show that the frequently observed overexpression of CSN6 in cancer promotes cancer stemness through the pathway depicted in our model (Fig. S2). Significantly, we discovered a negative relationship between CSN6 and TRIM21 in controlling OCT1 homoeostasis by regulating ubiquitin-mediated proteasomal degradation of OCT1. Our results provide insight into the consequence of CSN6–TRIM21 signalling on OCT1/ALDH1A1 expression during carcinogenesis and cancer progression.

CSCs are implicated in chemotherapy and radiotherapy resistance, metastasis and relapse. The failure of therapies to target CSCs leads to treatment failure. CSN6 has the capability to promote the formation of spheres enriched in CSCs, suggesting that CSN6 overexpression may promote distant metastasis and confer resistance after chemotherapy. Our previous observation that CSN6 overexpression reduces the recurrence-free survival of CRC patients^23^ is probably, at least in part, due to the promotion of cancer stemness by CSN6. Three-dimensional culture systems called organoids can be maintained by niche factors that support the growth, expansion and differentiation of stem cells in vitro. We found that decreasing CSN6 expression via knockdown reduced the number and growth of CRC PDOs, supporting the role of CSN6 in regulating stemness during tumorigenicity. Further studies are required to determine whether this role in promoting organoid proliferation could be used for CSN6-targeted drug screening.

Aldehyde dehydrogenase 1 (ALDH1) expression is a stem cell biomarker in various types of cancers, including CRC,^39^ and can be used to detect CSC populations. Expression of ALDH1 signifies cells with tumour-initiating or CSC properties in malignancies. Cells with high ALDH1A1 levels have increased expression levels of vimentin, matrix metalloproteinase-2 (MMP2), MMP7 and MMP9, which are implicated in epithelial–mesenchymal transition and metastatic capabilities.^40^ Interestingly, CSN6 is able to promote the expression of ALDH1A1, a known CSC marker upregulated in cancer spheroids.^41^ Chromatin immunoprecipitation assays have characterised ALDH1A1 as a direct target of β-catenin activation. Given that CSN6 positively regulates β-catenin by regulating β-Trcp ubiquitination,^41^ CSN6 may also regulate ALDH1A1 expression through the β-catenin pathway. It is interesting to note that both pathways operate through CSN6’s regulatory impact on the proteasome-mediated degradation system. Our data add a layer of complexity to the role of the canonical β-catenin–ALDH1A1 regulatory axis in promoting CSC formation.

Silencing ALDH1A1 using small interfering RNA can sensitise taxane- and platinum-resistant ovarian cancer cell lines to chemotherapy, suggesting a strategy of targeting ALDH1A1 to sensitise drug-resistant cells to chemotherapy.^42^ In addition, ALDH1A1 inhibitors targeting stem cell characteristics have been developed,^43^ for example, CM37. Treatment with CM37 leads to DNA damage and ROS production. Given that the expression level of ALDH1 is significantly increased in CSN6-overexpressing cells, ALDH1A1 inhibitors may be further characterised to treat CSN6-overexpressing CRCs with metastasis or drug resistance. Further investigation is warranted.

Tripartite motif (TRIM) protein family members (numbering >70) have been implicated in various cellular functions, including cell proliferation, differentiation, development, apoptosis, antiviral activity, autophagy and oncogenesis.^44^ TRIM21 plays a pivotal role in immune activation during pathogen infection, but its cellular function remains unclear.^45^ Interestingly, CSN6 is able to decrease the steady-state expression of TRIM21 by enhancing TRIM21 ubiquitination, thereby promoting cancer stemness. The impact of CSN6 on enhancing TRIM21 ubiquitination is reminiscent of the observations of CSN6’s effect on promoting self-ubiquitination of the E3 ligase β-Trcp during the regulation of β-catenin stabilisation.^41^ Several studies have shown that TRIM21 can perform tumour-suppressor functions. For instance, TRIM21 can cause fatty acid synthase (FASN) ubiquitination and degradation.^46^ In addition, degraded FASN leads to a decrease in de novo lipogenesis and inhibition of tumour growth. In this regard, TRIM21 functions as a tumour suppressor, as FASN is overexpressed in various types of cancer. In addition, TRIM21’s function is associated with autoimmune diseases, such as systemic lupus erythematosus (SLE) and Sjögren’s syndrome.^47,48^ Notably, patients with SLE or Sjögren’s syndrome have an increased risk for developing certain cancers, including non-Hodgkin’s lymphoma.^49^ In addition, TRIM21 interacts with endoglin,^50^ which is a prognostic marker in CRC^51^ and also act as a CSC marker in renal cell carcinoma.^52^ Future studies will need to address how the CSN6–TRIM21 axis may impact these signalling pathways to promote tumorigenesis.

OCT1 is known to regulate normal cell and CSC function.^33^ Loss of OCT1 in the colon was associated with restricted tumorigenicity.^43^ High OCT1 protein levels are correlated with the frequency of CD24 (low)/CD44 (high) cancer-initiating cells in primary malignant tissue.^33^ OCT1 is a transcription factor for ALDHs.^27^ Importantly, we show that CSN6 can reduce TRIM21-mediated OCT1 ubiquitination to regulate ALDH1A1 mRNA expression, thereby establishing the CSN6–TRIM21–OCT1–ALDH1A1 stemness-promoting axis.

Our findings in animal experiments indicate that the effect of this axis on promoting cell stemness can be recapitulated in vivo. In validation of the relevance of our findings to human cancer, our Kaplan–Meier analysis indicated that CRC patients with High CSN6 and Low TRIM21 expression exhibit the poorest overall survival. In addition, a high percentage of CRC patients exhibit a positive correlation between CSN6 and ALDH1A1 expression. ALDH1A1 not only can be used as a marker for stem cells but also regulates cellular functions involved in self-renewal and resistance to drugs and radiation. Our discovery implies that CSN6 overexpression may lead to drug and radiation resistance during tumorigenesis, thereby affecting survival. The involvement of high ALDH expression in resistance to several cytotoxic drugs, including cyclophosphamide and its analogues, doxorubicin, cisplatin, arabinofuranosyl cytidine, temozolomide and taxanes, is well documented.^53^ Targeting ALDH with a specific inhibitor could be a useful strategy for overcoming CSN6-mediated drug resistance issues in cancers.

In summary, we performed mechanistic studies illustrating the role of CSN6-facilitated TRIM21 ubiquitination in enhancing ALDH1A1 expression via the OCT1 transcription factor, lending support to the means by which CSN6 activity can lead to the initiation of cancer stemness. By regulating the TRIM21–OCT1–ALDH1A1 pathway through impacting on the ubiquitination process, CSN6 governs stemness, which is critical during CRC tumorigenesis. Taken together, our results allow the conclusion that CSN6 is an oncogene acting on cancer stemness signalling molecules, including TRIM21, OCT1 and ALDH1A1, and we propose that these molecules could be useful markers for cancers and are targets for anticancer drug development to improve the efficacy of chemotherapy and hinder tumour recurrence.

In conclusion, we validate a pathway for cancer stemness regulation involving ALDH1A1 levels through the CSN6–TRIM21 axis, which may be utilised as CRC molecular markers and be targeted for therapeutic intervention in cancers.

## Supplementary information

Supplementary file

## Data Availability

The data sets used and analysed during this study are available from the corresponding author on reasonable request.
