# Correction: CSN6–TRIM21 axis instigates cancer stemness during tumorigenesis

**DOI:** 10.1038/s41416-020-0977-5

**Published:** 2020-07-09

**Authors:** Baifu Qin, Shaomin Zou, Kai Li, Huashe Wang, Wenxia Wei, Boyu Zhang, Lishi Xiao, Hyun Ho Choi, Qin Tang, Dandan Huang, Qingxin Liu, Qihao Pan, Manqi Meng, Lekun Fang, Mong-Hong Lee

**Affiliations:** 1 Guangdong Provincial Key laboratory of Colorectal and Pelvic Floor Disease, The Sixth Affiliated Hospital of Sun Yat-sen University, 510655 Guangzhou, China; 2 Guangdong Research Institute of Gastroenterology, The Sixth Affiliated Hospital of Sun Yat-sen University, 510655 Guangzhou, China; 3 Department of Colorectal Surgery, The Sixth Affiliated Hospital of Sun Yat-sen University, 510655 Guangzhou, China

**Keywords:** Cancer stem cells, Colorectal cancer, Ubiquitylation

Correction to: *British Journal of Cancer* (2020) **122**, 1673–1685; 10.1038/s41416-020-0779-9, published online 30 March 2020

The original version of this article contained an error in Fig. 1f. The caption incorrectly listed ‘Csn6 high and Aldh1a1 low (*n* = 59)’ as ‘Csn6 low and Aldh1a1 low (*n* = 59)’, and ‘Csn6 high and Aldh1a1 high (*n* = 120)’ as ‘Csn6 low and Aldh1a1 high (*n* = 120)’. This correction has been made to the caption and a corrected version of the Figure is below.

**Fig. 1:**
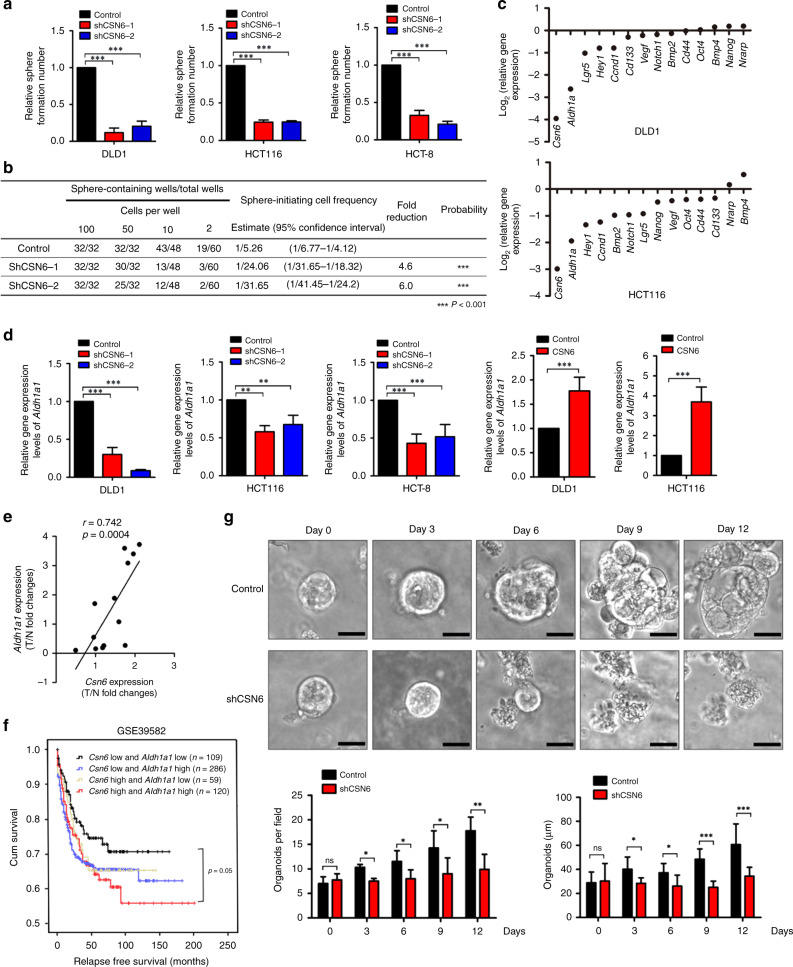
CSN6 is required for sphere formation and initiates stemness through ALDH1A1. **a** Sphere-formation assay of DLD-1, HCT116 and HCT-8 cells carrying scrambled or CSN6-specific shRNA. **b** DLD-1 cells carrying scrambled or CSN6-specific shRNA were dissociated into a single-cell suspension, seeded in 96-well plates with an ultra-low attachment surface at a density of 2, 10, 50 or 100 cells per well and cultured for 12 days. The frequency of sphere-initiating cells was estimated using the ELDA software. **c** Quantitative RT-PCR analysis was performed to measure the mRNA levels of stem cell markers (*Aldh1a1*, *Lgr5*, *Cd133* and *Cd44*), embryonic stem cell components (*Nanog* and *Oct4*), WNT pathway components (*Vegf* and *Ccnd1*), Notch pathway components (*Notch1*, *Hey1* and *Nrarp*) and BMP family genes (*Bmp2* and *Bmp4*) in DLD-1 cells and HCT116 cells carrying scrambled or CSN6-specific shRNA. **d** Quantitative RT-PCR analysis was performed to measure the mRNA levels of Aldh1a1 in DLD-1, HCT116 and HCT-8 cells with CSN6 knockdown or CSN6 overexpression. **e** Quantitative RT-PCR analysis was performed to measure the mRNA levels of colorectal cancer and adjacent colorectal tissues. The levels of Csn6 were positively correlated with the expression of *Aldh1a1* at mRNA levels in 13 pairs of human colorectal carcinomas (T) with matched normal tissues (N). **f** Kaplan–Meier survival curves of relapse-free survival time based on *Csn6* and *Aldh1a1* expression in CRC tissues. *P < 0.05, **P < 0.01 and ***P < 0.001. **g** Knockdown of CSN6 affected patient-derived tumour organoid (tumour PDO) growth. The morphology of the organoids is shown. The number of organoids growing to a size of >25 μm was calculated. Scale bars, 25 μm.

