# Supplementary material for: CSN6–TRIM21 axis instigates cancer stemness during tumorigenesis
Source: Br J Cancer. 2020 Mar 30;122(11):1673–85. doi: 10.1038/s41416-020-0779-9 (PMC7250844; doi:10.1038/s41416-020-0779-9)
Supplement: Supplementary file 1 — Supplementary file [file 41416_2020_779_MOESM1_ESM.docx]

**Supplementary Tables**

**Table S1. Primers for qPCR**

| Gene | Forward primer sequence | Reverse primer sequence |
| --- | --- | --- |
| *Csn6* | TCATCGAGAGCCCCCTCTTT | CCAATGCGTTCCGCTTCCT |
| *Trim21* | GTCCTGGAAAGGAGTGAGTCC | CTGAAAGTATCAGCCACGGATT |
| *Aldh1a1* | TGTTAGCTGATGCCGACTTG | TTCTTAGCCCGCTCAACACT |
| *Lgr5* | CTTCCAACCTCAGCGTCTTC | TTTCCCGCAAGACGTAACTC |
| *Cd133* | AGTCGGAAACTGGCAGATAGC | GGTAGTGTTGTACTGGGCCAAT |
| *Cd44* | CTGCCGCTTTGCAGGTGTA | CATTGTGGGCAAGGTGCTATT |
| *Oct4* | CCTGAAGCAGAAGAGGATCA | CCGCAGCTTACACATGTTCT |
| *Nanog* | TTTGTGGGCCTGAAGAAAACT | AGGGCTGTCCTGAATAAGCAG |
| *Ccnd1* | GCTGCGAAGTGGAAACCATC | CCTCCTTCTGCACACATTTGAA |
| *Vegf* | AGGGCAGAATCATCACGAAGT | AGGGTCTCGATTGGATGGCA |
| *Notch1* | GGACGTCAGACTTGGCTCAG | ACATCTTGGGACGCATCTGG |
| *Hey1* | CGGCTCTAGGTTCCATGTCC | GCTTAGCAGATCCCTGCTTCT |
| *Nrarp* | GCGTTGTGAAGGCAACAGAG | GGGAGGCTAAAAAGGGGCAA |
| *Bmp2* | TTCGGCCTGAAACAGAGACC | CCTGAGTGCCTGCGATACAG |
| *Bmp4* | ATGATTCCTGGTAACCGAATGC | CCCCGTCTCAGGTATCAAACT |

**Table S2. Correlation between expression of CSN6 and clinicopathological features of colorectal cancer patients**

|  | Low CSN6 | High CSN6 | p value^a^ |
| --- | --- | --- | --- |
| Gender |  |  | 0.460 |
| Male | 65 (54.2) | 72 (49.0) |  |
| Female | 55 (45.8) | 75 (51.0) |  |
| Median age |  |  | 0.712 |
| <59 years | 55 (45.8) | 64 (43.5) |  |
| ≥59 years | 65 (54.2) | 83 (56.5) |  |
| Histological grade |  |  | 0.860 |
| G1 | 4 (12.9) | 7 (14.0) |  |
| G2 | 21 (67.7) | 31 (62.0) |  |
| G3 | 6 (19.4) | 12 (24.0) |  |
| pT status |  |  | 0.096 |
| T1 | 7 (5.8) | 2 (1.4) |  |
| T2 | 19 (15.8) | 16 (10.9) |  |
| T3 | 93 (77.5) | 126 (85.7) |  |
| T4 | 1 (0.8) | 3 (2.0) |  |
| pN status |  |  | 0.306 |
| N0 | 72 (60.0) | 98 (66.7) |  |
| N1 | 48 (40.0) | 49 (33.3) |  |
| pM status |  |  | 0.034 |
| M0 | 112 (93.3) | 125 (85.0) |  |
| M1 | 8 (6.7) | 22 (15.0) |  |
| Clinical stage |  |  | 0.015 |
| I | 20 (16.7) | 12 (8.2) |  |
| II | 48 (40.0) | 71 (48.3) |  |
| III | 44 (36.7) | 42 (28.6) |  |
| IV | 8 (6.7) | 22 (15.0) |  |

NOTE: All data are no. of patients (%).

^a^p values were calculated in SPSS16.0 using a chi-square test. p values <0.05 were considered to indicate statistical significance.

**Supplementary figures**

**Figure S1 CSN6 is required for sphere formation and positively related with ALDH1A1**


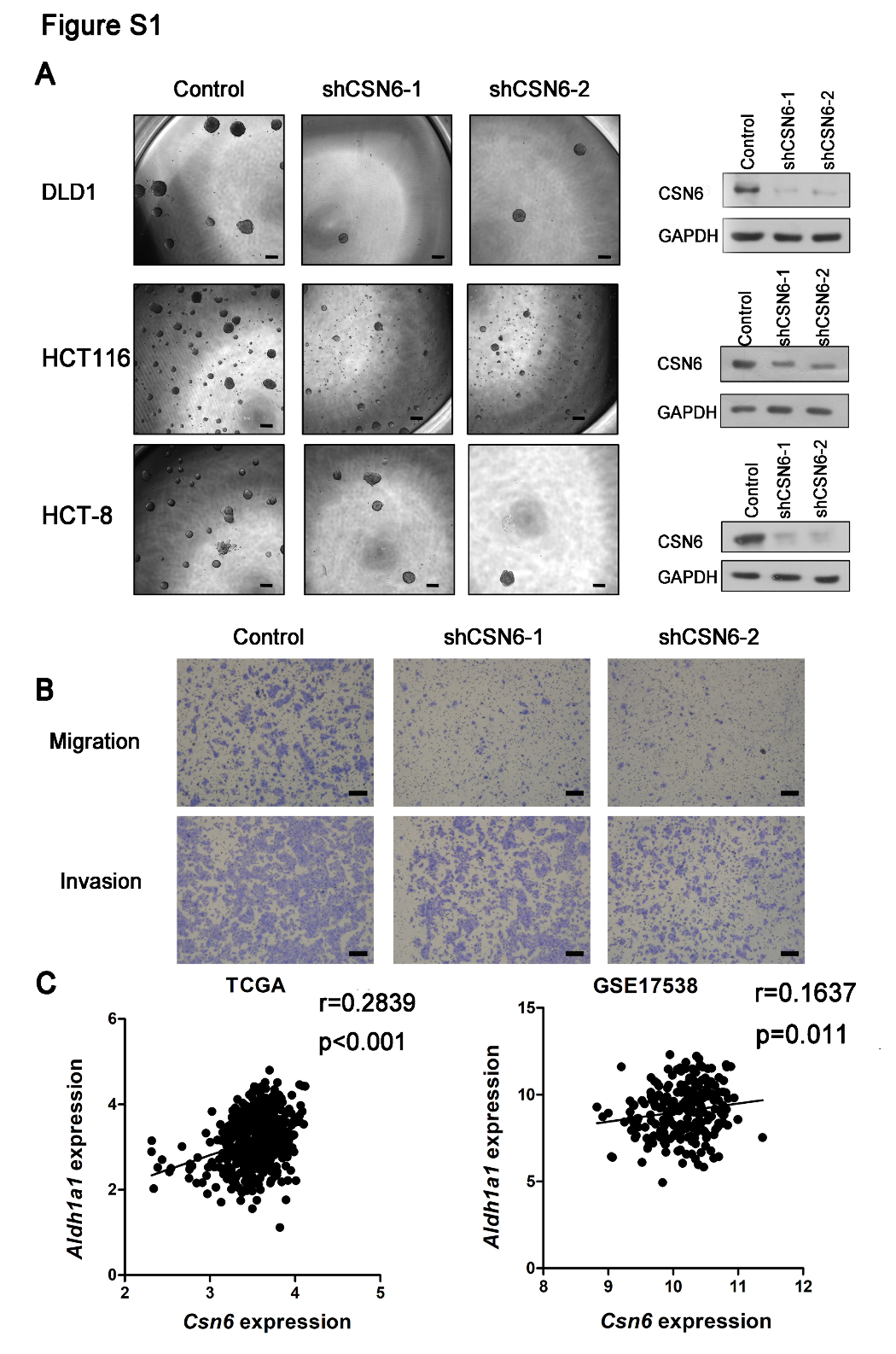


(A) Sphere formation assay of DLD-1 cells, HCT116 cells and HCT-8 cells carrying scrambled or CSN6-specific shRNA. The scale bars represent 200 μm.

(B) Migration and invasion assays of DLD-1 cells carrying scrambled or CSN6-specific shRNA. The scale bars represent 200 μm.

(C) Correlation between the *Csn6* mRNA level and *Aldh1a1* mRNA level in CRC in the TCGA and GSE17538 datasets.

^**^P<0.01 and ^***^P<0.001.

**Figure S2 Model showing the role of CSN6 overexpression in promoting cancer stemness**
